# Different Pitch Configurations Constrain the Playing Tactics and the Creation of Goal Scoring Opportunities during Small Sided Games in Youth Soccer Players

**DOI:** 10.3390/ijerph181910500

**Published:** 2021-10-06

**Authors:** Joaquín González-Rodenas, Rodrigo Aranda-Malavés, Andrés Tudela-Desantes, Pedro de Matías-Cid, Rafael Aranda

**Affiliations:** 1 Centre for Sport Studies, Rey Juan Carlos University, 28942 Madrid, Spain; 2 Department of Physical Education and Sports, University of Valencia, 46010 Valencia, Spain; Rodrigo.Aranda@uv.es (R.A.-M.); Andres.Tudela@uv.es (A.T.-D.); Pedro.Matias@uv.es (P.d.M.-C.); 3 Doctoral School, Catholic University San Vicente Mártir, 46001 Valencia, Spain

**Keywords:** non-linear pedagogy, constraints led approach, sport pedagogy, practice design, football training

## Abstract

The aim of this study was to explore the tactical effects of different pitch configurations on the collective playing tactics and the creation of goal scoring opportunities (GSO) during small sided soccer games (SSG) in youth players. A total of 22 players performed a 7 vs. 7 + 1 floater (including goalkeepers) under three different pitch configurations (“Standard”, 53 × 38 m; “Long”, 63 × 32 m; and “Wide”, 43 × 47 m). Eleven tactical indicators related to the development and the end of the team possessions were evaluated by systematic observation. Friedman tests (non-parametric ANOVA for repeated measures) revealed that the long and wide configurations produced more counterattacks (*p* = 0.0028; ES = 0.3), higher offensive penetration (*p* = 0.007; ES = 0.41), and more GSO (*p* = 0.018; ES = 0.30) than the standard format. Regarding the creation of GSO, the wide configuration produced more assists in the form of crosses than the long and standard formats (*p* = 0.025; ES = 0.31), more utilization of wide subspaces to assist the final player (*p* = 0.022; ES = 0.35), more number of headers as the final action (*p* = 0.022; ES = 0.32), and less assists in the form of passes in behind the defense (*p* = 0.034; ES = 0.28), than the long configuration. The modulation of the pitch configuration during SSG produced different tactical demands, requiring players to implement different tactical solutions to create GSO.

## 1. Introduction

The key offensive aim of soccer is to disorder the defensive organization of the opposing team to achieve goal scoring opportunities (GSO) and goals. During that process, the last actions such as the assist and the final shot are decisive and both depend on the spatial–temporal relationship between the penultimate and the last player as well as the interaction between the offensive and defensive organizations [1,2,3,4]. Due to its complexity and importance, one of the most challenging duties of professional soccer clubs is to develop or recruit players who have the capacity to score or create high amounts of GSO. In fact, recent studies have observed that goal scorers and players with high quantity of assists demonstrated to have higher levels of cognitive functions such as creativity and working memory, compared with other players [5,6]. In addition, the study of Kempe and Memmet [7] observed that showing high creativity in the last two actions before the actual shot on goal proved to be the best predictor for game success in elite soccer.

Regarding the tactical development of GSO and goals, existing literature found that the actions that led to goal were predominantly originated in central and advanced areas of the opposing half, while the crosses have been shown to represent around 30–40% of the goal assists [8]. Furthermore, the majority of goals and GSO seem to be produced by collective plays, so that the individual actions produced less than 20% of them [9,10,11]. For instance, the study of González-Rodenas et al. [12] analyzed 1172 GSO from top European teams and observed that crosses comprised the 19.6% of penultimate actions prior to GSO, while passes comprised the 62.4% and individual actions the 9.1%.

Nevertheless, the tactical actions performed prior to goals or GSO are influenced by the interaction with the opposing team [2,3,13] In this regard, González-Rodenas et al. [4] observed that crosses were more frequent against organized defenses, while passes in behind the defense or actions as dribbling or running with the ball had a greater percentage of goals against circumstantial defenses. Regarding the final action, 70.1% of the goals were scored by using only one contact to the ball in organized defenses but 46.6% in circumstantial defenses. These tactical facts suggest that players should be able to adapt to the defensive context and interact with their teammates to choose the best solution in order to disrupt the opposing team and create a GSO or a goal.

However, despite the growing emergence of research about small sided games (SSG) in soccer [14], there is a lack of studies that specifically focus on how different types of SSG can influence the way GSO and goals are achieved in training. In recent years, the analysis of SSG in soccer has focused on physiological variables [15], motion analysis [16,17], collective behavior [18,19], and technical and tactical performance of players [20,21,22].

Consequently, it seems necessary to explore training methods to optimize the creation of GSO in youth soccer. In this sense, the constraints-led approach based on non-lineal pedagogy has emerged as an optimal framework to create representative learning designs in collective sports [23]. Under this framework, coaches should manipulate task constraints during the SSG such as space, time, rules, goals, or number of players in order to create learning environments where players can interact with their teammates and opponents to explore opportunities for action and adapt to the specific tactical context. This manipulation of task constrains encourage the player’s co-adaptive and exploratory behavior to search for effective solutions, embracing problem-solving situations [24].

One of the easiest constraints for coaches to modify in SSG is the space. In this sense, the effect of the manipulation of the space size on physical and technical demands has been analyzed in multiple studies about SSG [25]. However, no study to date has analyzed the effect of modifying the pitch configuration on the playing tactics and creation of GSO in soccer. In this sense, Coutinho et al. [19] analyzed the effect of different pitch configuration on the physical demands and movement behavior in young players, concluding that using different pitch configurations might help players to improve their ability to identify the most relevant cues that support the emergence of functional behaviors. Likewise, Folgado et al. [26] observed that a longer pitch configuration (40 × 30 m) during a 4 vs. 4 + GKs game registered more distance covered at high intensities, more passes, and dribbles than a wider format (30 × 40 m), which had more lateral passes and shots and a wider team positioning.

According to this background, our hypothesis is that the design of SSG with different pitch configurations (i.e., wide pitch vs. long pitch vs. standard pitch) may modulate the emergence of collective tactical behaviors and encourage players to explore different ways to build and create GSO. Therefore, the aim of this study was to check the tactical effects of different pitch configurations on the collective playing tactics and the creation of GSO during SSG in youth players.

## 2. Materials and Methods

### 2.1. Sample

A total of 22 elite youth players (age: 13.80 ± 0.58, years of experience: 6.30 ± 0.95) from an American professional soccer club participated in the study. The participants, parents, and the club were informed about the research procedures and provided written informed consent. This study followed the ethical standards for study in humans as outlined in the Declaration of Helsinki. The sample comprised 296 offensive possessions according to the definition of Pollard and Reep [27] that teams performed during the small sided soccer games.

### 2.2. Small Sided Games

**Figure 1:**
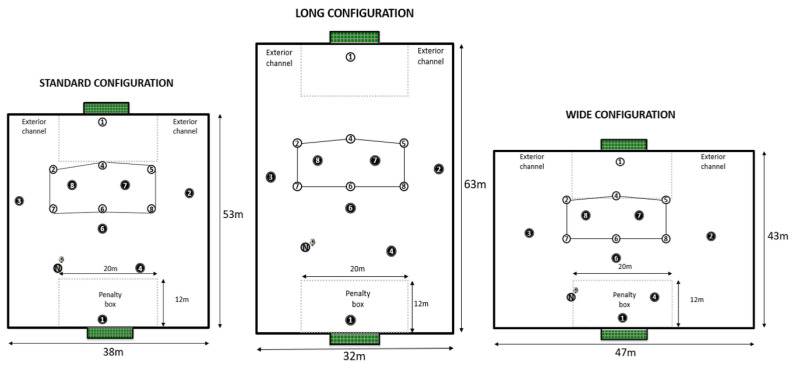
Different pitch configurations and field sizes of the small sided soccer games.

The players performed the same type of SSG (7 vs. 7 + 1 floater, including goalkeepers) under three different pitch configurations (“Standard”, 53 × 38 m; “Long”, 63 × 32 m; and “Wide”, 43 × 47 m) (Figure 1). The SSG were performed two times per week for a period of 3 weeks as part of the normal routine training sessions of the U14 team [28], following a standardized warm-up protocol consisting of 5 min of dynamic mobility and 5 min of technical actions. All the training sessions took place in the spring season, during the same hours (7.00 p.m.), on the same artificial turf surface. The SSG were recorded with one digital camera (Panasonic HC-V180) from an aerial perspective (10 m above the ground) to capture entirely the collective movements of both teams, following the routine way of filming of the team’s coaching staff.

The order in which the SSG formats were performed during the course of the training sessions was randomized (order in week 1: standard, long, and wide; order in week 2: long, wide, and standard; order in week 3: wide, standard, and long).

Table 1 shows the main features of the SSG conducted in this study. The SSG design included official goals and goalkeepers, and the coach did not provide direct instructions about how to solve the tactical situations or to adapt to the different spatial constraints. In addition, the teams had identical tactical objectives and tactical formations during the SGG

The SSG implemented were part of the real tactical training of the team during the spring season. To contextualize, the team’s game model was based on building-up from the back, having long ball possessions to disorder the opposing team and creating GSO. This style of play required high passing accuracy and speed but also high amount of patience to decide when and where to break lines of the defensive team to reach the opposing goal. Within this game model, the team’s structure and dynamic encouraged the wingers (forwards in our design) to play in interior channels near the midfielders to create offensive superiority in central spaces. Meanwhile, the full backs were encouraged to advance through the wide channels to reach offensive zones, playing an important role in the attacking process to create GSO.

Due to this tactical context, the objective of the coaching staff was to design representative learning designs [29] for players to experience different spatial scenarios where to dominate the ball possession and to create GSO, while they could interact with their teammates and opponents in their real positions.

**Figure 2:**
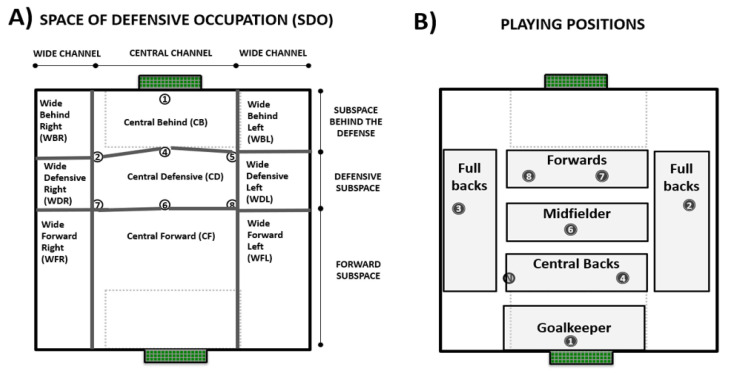
(**A**) Space of defensive occupation (SDO) of the defensive team [42]. This spatial organization is defined by Gréhaigne [43] as the “space that is constituted by the positions of the players located, at a given moment, in the periphery of a team in play, except the goalkeeper”. This space is subdivided into 9 different subspaces that define the level of penetration and width in relation to the opponent (adapted from previous studies, [42,43,44]). These subspaces are dynamic and change every second depending on the positioning on the opposing players. (**B**) Playing positions of players considered in this study.

Therefore, the team’s system during the SSG (Figure 2B) tried to reproduce the actual game model in the attacking moment, having:

- One goalkeeper and one central back that should lead the process of building-up from the back.
- One midfielder that had the role of connecting the build-up with the finishing process.
- Two forwards that should occupy interior channels to create superiority in the build-up process and to play a relevant role in the finishing process.
- Two full backs that, in addition to build-up, had an important role reaching offensive zones and creating GSO.
- Additionally, one offensive in-floater was added to, according to the existing scientific literature, increase the passing possibilities and interactions of the attacking team [30,31] as well as to encourage the defensive team to stay compact and to prioritize the protection of the goal, [32], as we can observe in Figure 2A.

It is important to mention that although teams were structured in specific positions and roles, players were free to make their own decisions, movements, and actions to solve the different tactical situations during the SSG, in interaction with their teammates.

Regarding the pitch sizes and configurations selected, the coaching staff decided to compare the standard configuration with an extra-wide and an extra-long format, in order to expose players to different spatial constraints. The final design was based on the work of previous studies [19,26,33] that suggested that modifying the pitch configurations by changing the width and length affects team’s spatial and temporal interaction. Although the pitch configuration was changed, the area per player was maintained stable across the three formats. This size (132 m^2^ per player) was established considering previous literature on SSG [14,25] since most of the studies used pitch sizes that involved a range between 100 and 150 m^2^ per player.

Furthermore, in order to promote the build-up of GSO from the back, the SSG did not have throw-ins, corner kicks, or free kicks, so that all the restarts were initiated as goal kicks by the team in possession of the ball.

Finally, the teams were balanced according to the technical and tactical ability of the players in order to have very competitive games and teams were maintained throughout the duration of the study.

### 2.3. Performance Analysis

The study was based on systematic observation [34]. Eleven tactical dimensions (Table 2, Table 3 and Table 4) related to the offensive team possessions were analyzed using the REOFUT theoretical framework [35] that provides a valid and reliable tool to analyze multiple tactical and technical dimensions related to the start, development, penultimate, and last action of teams’ possessions as well as their association with achieving offensive performance [9,12].

For the analysis, a soccer coach/researcher experienced in match performance analyzed each possession post-event as many times as necessary. The Lince software [36] was used to code and register the data. The reliability of data was calculated by the intra and inter-observer agreement (Cohen’s Kappa). For this purpose, the principal researcher, and another researcher with broad experience in soccer tactical analysis (UEFA Pro Soccer Coach and PhD in Sport Sciences) evaluated 100 random possessions (33% of the sample). This analysis showed good and very good level of reliability according to Altman criteria [37] (inter-observer kappa coefficient = 0.82–1.00; intra-observer kappa coefficient = 0.84–1.00).

**Table 2:** Description and categories for the dimensions related to the start and development of the team possession.

**1-Possession type:** way to start a team possession according to if the ball is in play or out of play. Two categories were considered: (**A**) **Transition play:** when a player gains the possession of the ball by any means other than from a player of the same team with the ball in play. (**B**) **Restart:** when a player initiates the team possession after the ball was out of play. In these SSG, all the restarts were taken as goal kicks by the goalkeeper.
**2-Type of attack:** degree of offensive directness and elaboration during the offensive process [38,39,40,41]. Two categories were considered: (**A**) **Organized attack:** (a) the possession starts by winning the ball in play or restarting the game; (b) in this type of team possession the opposing team is organized defensively or is able to re-organize its collective defensive system during the possession. (**B**) **Counterattack:** the possession starts by winning the ball in play; the opponent is not organized defensively and is not allowed to re-organize their collective defensive system during the team possession; the progression towards the goal attempts to utilize a degree of imbalance right from start to the end with high tempo [39]; the circulation of the ball takes place more in depth than in width, using a high percentage of penetrative passes. The intention of the team is to exploit the space left by the opponent when they were attacking.
**3-Possession width:** occupation of the interior and/or exterior channels within the space of defensive occupation of the opponent (SDO) [42] (Figure 2A). Three categories were considered: (**A**) **Minimum width:** During the possession, the ball moves through one channel of the SDO. **Medium width:** During the possession, the ball moves through two channels of the SDO© **Maximum width:** During the possession, the ball moves through the three channels of the SDO.
**4-Passes per possession:** number of passes performed by the offensive team during the possession.

**Table 3:** Description and categories for the dimensions related to the penultimate and last action of the possession.

**5-Penultimate action:** technical-tactical action performed immediately before the final action that allows the final player to have the opportunity of shooting at goal. This action may be performed by the same player that shoots at goal (individual action) or by a teammate that pass the ball to the final player (collective play). Four categories were considered: (**A**) **Individual action:** the final player receives the ball without having a scoring opportunity but he succeeds in creating one by means of an individual action such as dribbling, running with the ball, collecting a free ball, or shooting from distance (the players shoot from outside the penalty box (Figure 1). (**B**) **Collective play:** the penultimate player in the team possession performs a pass that allows the last player to have an immediate scoring opportunity. This category has three sub-categories. (**C**) **b.1 Pass in behind the defense:** pass from central channels of the field that breaks the opposing defensive line and allows the receiver to have an immediate scoring opportunity in front of the goalkeeper. (**D**) **b.2 Cross:** pass performed from the wide channels of the field in the opposing half (Figure 1) towards the penalty box [28] that allows the receiver to have an immediate scoring opportunity. (**E**) **b.3. Goal pass:** the final player receives an assist in form of a pass (different from a pass in behind and cross) from a different player that allows him to have an immediate scoring opportunity.
**6-Penultimate player:** specific position of the player that performs the penultimate action. Five categories were considered **(A) Goalkeeper, (B) Central defender, (C) Full back, (D) Central midfielder, (EF) Forward**
**7-Penultimate invasive subspace:** area within the space of defensive occupation (SDO) [42] of the opponent where penultimate action is done (Figure 2A). Three categories were considered: (**A**) Subspaces behind the defense (WBL, CB, WBR). (**B**) Defensive subspace. (WDL, CD, WDL). (**C**) Forward subspace (WFR, CF, WFL).
**8-Last player:** specific position of the player that performs the last action. Five categories were considered **(A) Goalkeeper, (B) Central defender, (C) Full back, (D) Central midfielder, EF) Forward.**
**9-Last action:** technical action performed by the last player who had the GSO. Three categories were considered: (**A**) **Shoot with 1 contact:** the possession ends with a shot on goal by means of a single contact. (**B**) **Shoot with 2 or more contacts:** the possession ends with a shot on goal by means of two or more contacts. (**C**) **Header:** the final player shoots at goal by heading the ball.
**10-Offensive performance:** degree of offensive success of the possession, based on the degree of penetration over the opposing team and the achievement of GSO and goals. Three categories were considered: (**A**) **Scoring opportunity:** the possession ends with a clear chance of scoring a goal during team possession (goals are included). This includes all shots and all the chances of shooting that one player has inside the penalty box (the player is facing the goal, there is not any opponents between him and the goal, and he has enough space and time to make a playing decision). The shots taken from outside the penalty box are considered GSO when the ball passes near the goal (2 m or less with respect to the goal). (**B**) **Offensive penetration:** the team possession achieves to beat the forwards and midfielders’ lines of the opponent and face directly the defensive line during the offensive sequence. However, the possession ends without creating any GSO. The player(s) facing the defensive line has/have enough time and space to perform intended actions on the ball at the moment of receiving the ball. (**C**) **No offensive penetration:** the team possession does not achieve disorder and beat the forwards or midfielders’ lines of the opposing team during the offensive sequence.
**11-Last invasive zone:** area within the space of defensive occupation (SDO) [42] of the opponent where last action is performed (Figure 2A). Three categories and nine sub-categories were considered: (**A**) Subspaces behind the defense (WBL, CB, WBR). (**B**) Defensive subspace. (WDL, CD, WDL). (**C**) Forward subspace (WFR, CF, WFL).

### 2.4. Statistical Analysis

Data was transcribed to a database created in the SPSS 20.0 program (SPSS, Chicago, IL, USA). All results are reported as mean, standard deviations (mean ± SD), and medians. Data represents the mean percentage of playing tactics or scoring opportunities implemented or created by the teams in each pitch configuration format. To show the differences between formats in the offensive penetration and creation of GSO, boxplot graphs were displayed to assess and compare the shape, central tendency, and variability of the sample.

The non-normality of the data was verified using the Shapiro–Wilk test. The Friedman test (non-parametric ANOVA for repeated measures) was used to detect tactical differences (dependent variables) between the three pitch configurations (independent variables). Dunn–Bonferroni post hoc tests were carried out to determine the differences between pairs. The confidence interval was set at 95%. The effect sizes were calculated using the Kendall’s W (coefficient of concordance), (0.1–0.3 = small effect; 0.3–0.69 = moderate effect; 0.69–1.0 = large effect size).

## 3. Results

### 3.1. Collective Playing Tactics

Table 4 shows the playing tactics implemented by the teams during the SSG with different pitch configurations. The long configuration registered higher percentage of counterattacks (34.6 ± 12.4%) than the standard one (20.7 ± 13.4%) (*p* ˂ 0.05) and both long and wide had more team possessions that started by means of transition play than the standard format (60.8 ± 15.9% and 62.2 ± 12.4% vs. 45.7 ± 18.8%, respectively) (*p* ˂ 0.05). Additionally, the wide configuration registered less team possessions that achieved reduced width (10.9 ± 9.9%) in comparison with the standard format (27.3 ± 13.5%) (*p* ˂ 0.05).

**Table 4:** Comparison of playing tactics between the three different pitch configurations.

Category	Standard	Long	Wide	*p* *	ES ^#^
	**Mean ± SD (Median)**	**Mean ± SD (Median)**	**Mean ± SD (Median)**		
Type of attack					
Counterattack	20.7 ± 13.4 (17.1)	34.6 ± 12.4 a (33.3)	27.4 ± 12.6 (26.8)	**0.028**	**0.30**
Positional attack	79.3 ± 13.4 (82.8)	65,3 ± 12.4 (67.7) ^a^	72.6 ± 12.4 (73.2)	**0.028**	**0.30**
Passes per possession	5.0 ± 0.8 (4.9)	4.2 ± 1.2 (4.2)	4.53 ± 0.87 (4.6)	0.105	0.18
Transition play	45.7 ± 18.8 (46.4)	60.83 ± 15.95 (60.0) ^a^	62.2 ± 12.4(63.5) ^a^	**0.010**	**0.39**
Restart	54.4 ± 18.8 (54.0)	39.16 ± 17.21 (40.0) ^a^	37.7 ± 12.4(36.4) ^a^	**0.010**	**0.39**
Offensive width					
Maximum width	29.7 ± 11.8 (29.3)	35.5 ± 19.6(36.6)	45.8 ± 17.0 (44.4)	0.083	0.21
Medium width	42.9 ± 17.7 (45.0)	37.8 ± 13.9 (38.7)	43.3 ± 18.1 (43.7)	0.517	0.06
Reduced width	27.3 ± 13.5 (25.0)	26.7 ± 17.8 (19.4)	10.9 ± 9.9 (12.5) ^a^	**0.018**	**0.34**
Offensive penetration	49.1± 17.7 (50.0)	63.0 ± 15.2 (63.3) ^a^	71.7 ± 17.4 (70.8) ^a^	**0.007**	**0.41**
Scoring opportunity	26.9 ± 14.6 (28.6)	40.9 ± 21.0 (42.7) ^a^	43.2 ± 17.8 (43.6) ^a^	**0.026**	**0.30**

* Friedman Test. Values in bold indicate significant differences between pitch configurations. ^#^ Effect size calculated using the Kendall’s W (coefficient of concordance; 0.1–0.3 = small effect; 0.3–0.69 = moderate effect; 0.70–1.0 = large effect size. ^a^ = significantly different (*p* ˂ 0.05) from the “standard” configuration.

### 3.2. Offensive Performance

**Figure 3:**
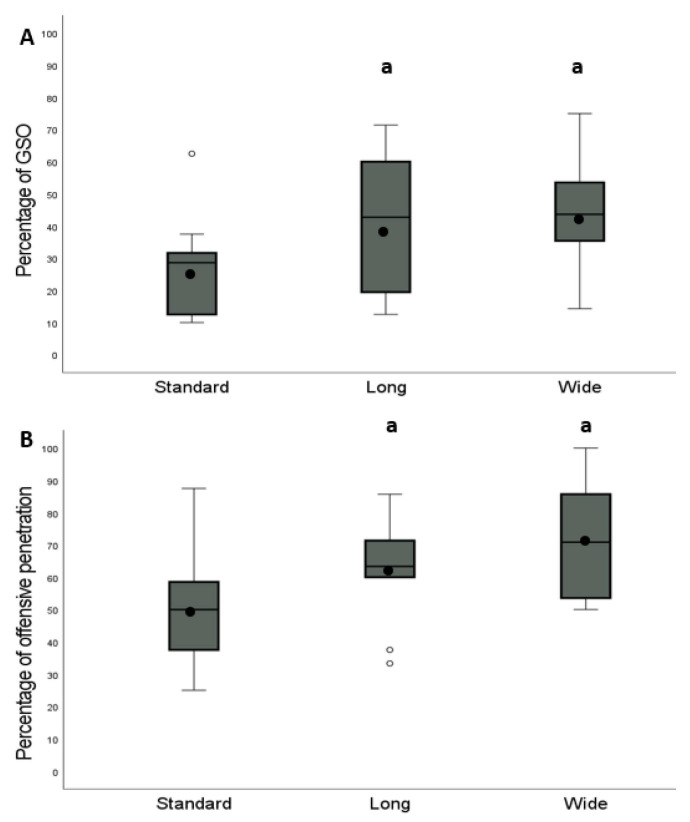
Box plot of the percentage of team possession that achieved (**A**) goal scoring opportunities and (**B**) offensive penetration during the different pitch configuration of the SSG. The box indicates the 25th and 75th quartiles and the central line is the median. The ends of the whiskers are the 2.5% and 97.5% values. Values outside the range of the whiskers are extreme values. Mean is represented with a black dot. a = significantly different (*p* ˂ 0.05) from the “standard” configuration.

Figure 3 shows that both long (63.0 ± 15.2%) and wide (71.7 ± 17.4%) formats achieved more offensive penetration than the standard format (49.1 ± 17.7%) (*p* ˂ 0.05). In addition, both long (40.9 ± 21.0%) and wide (43.2 ± 17.8%) were more effective in creating GSO than the standard configuration (26.9 ± 14.6%) (*p* ˂ 0.05).

### 3.3. Goal Scoring Opportunities

Table 5 shows the playing tactics implemented when creating GSO. No significant differences were found for all the dimensions except for the offensive width, where the wide format registered lesser frequency of team possessions that had reduced width (10.9 ± 14.8%) than the long format (30.1 ± 31.8%) (*p* ˂ 0.05).

Regarding the penultimate action when creating GSO (Table 6), the wide configuration produced more assists in the form of crosses (43.0 ± 25.1%) than the long (13.3 ± 20.5%) and standard formats (16.6 ± 28.6) (*p* ˂ 0.05). In addition, the wide configuration had more utilization of wide subspaces (65.3 ± 20.8%) to assist the final player than the long configuration (29.2 ± 22.0%) (*p* ˂ 0.05). Finally, the long configuration registered more passes in behind the defense (30.8 ± 31.5 than the wide format (12.8 ± 24.0%) (*p* ˂ 0.05).

As for the last action when creating GSO, Table 7 shows that no significant differences were found for the final player and the final subspaces either in width or penetration level. As regards the last technical action, a greater number of headers was found in the wide configuration (16.6 ± 18.4%), in comparison with the long format (1.7 ± 5.8%).

## 4. Discussion

The aim of this study was to explore the tactical effects of different pitch configurations on the collective playing tactics and the creation of goal scoring opportunities during SSG in youth soccer players. Our research observed that manipulating the pitch configuration during SSG to make the field “longer” or “wider” can modulate some of the technical and tactical actions performed by players and teams to create GSO.

To our knowledge, this study is the first to analyze the tactical creation of GSO in SSG with different pitch configurations by means of observational methodology. This fact makes it difficult to compare our findings with other studies, since previous studies evaluated the effects of different pitch shapes on physical and physiological variables [45], as well as on collective team behaviors [19,33] and technical actions [26] However, some of their findings may be useful for the interpretation of our results.

First of all, both the long and the wide configurations created more ball transitions between teams in open play, more counterattacks, more offensive penetration, and more GSO than the standard format. These tactical aspects may be due to the change in the space constraints experienced by the players. For instance, the study of Coutinho et al. [19] observed that SSG with standard condition registered higher collective movement synchronization in both longitudinal and lateral directions, in comparison to other formats such as a sided configuration. Although the methodology used in the research of Coutinho et al. [19] is very different from our study, their findings could help in the interpretation of our results. In this regard, a possible higher collective synchronization in the standard format would create a more defensively organized scenario in which penetrating and creating GSO could be more difficult than in the other formats, in which the different spatial constraints may reduce the collective organization and create a more open context to break lines of the opponent.

For example, the long configuration offers a tactical context where the reduced spatial width may provoke teams trying to advance the opposing goal with more verticality. This scenario may cause more ball losses and changes in the ball possessions between teams in open play, but also it could contribute to creating more “attempts” to break opposing lines, which would explain the higher offensive penetration and number of GSO than the standard format. In addition, the long configuration increases the distance that teams need to cover to reach the opposing goal or to move back to defend the own goal. In fact, the study of Folgado et al. [26] observed that increasing the field’s length contributes to an increase in team’s length and the distances between the team’s centroids, which can create more space between the lines of the defensive team. This tactical constraint can increase the opportunities to perform counterattacks to exploit the space left behind by the opponent when trying to attack, which could lead to more offensive penetration and GSO than in the standard configuration.

As for the wide configuration, two main constraints can influence the modulation of the offensive process in comparison with other formats. On one hand, the wide format reduces the length of the field in comparison to the standard or long formats, which reduces the distance between goals and may make it easier to reach shooting areas, explaining the higher degree of offensive penetration and GSO. Our results agree with Folgado et al. [26] who observed more shots per player in a wider field (30 × 40 m) rather than in a standard one (40 × 30 m). On the other hand, this configuration allows the offensive team to have more space to progress in the wide channels, which makes it more demanding for the defensive team to move laterally and prevent the offensive penetration. In this sense, the study of Folgado et al. [26] observed that increasing the field’s width contributes to increases in the team’s width, which indicates that teams need to increase the distance between teammates to cover more space laterally, which can also help the offensive team penetrate through the interior subspaces of the defensive team. In addition to a higher team’s width, Coutinho et al. [19] observed that a sided pitch did not lead to a higher time spent synchronized compared to the standard configuration. These findings defend the idea that in wider fields, the coordination between teammates decreases, which could lead to more opportunities for the offensive team to penetrate and create GSO.

Regarding the team possessions that led to GSO, the main findings of our study revealed that the wide configuration created more GSO by crossing than the rest of the formats, as well as more headers as the final action than the long configuration. Meanwhile, the long format had a higher frequency of penultimate actions in the form of passing in behind the defense than the wide format.

These results indicate that players explored and implemented different solutions to achieve GSO during the different SSG. For example, the long configuration offers a tactical context where the defensive team may not only have more length but they could also leave more space between their defensive line and the goalkeeper. Under these conditions, the offensive team may have more opportunities to make runs in behind the defensive line to try to exploit this subspace and create GSO. Interestingly, Folgado et al. [26] observed that more elongated pitch elicited more distance covered at high intensities, higher number of forward passes, and a larger distance between the goalkeeper and the last defender. In this tactical context, teams seem to play more vertical in order to gain advantage of the unoccupied space between the last defender and the goalkeeper, which makes players explore a different way of creating GSO than in a wider field, where the spatial constraints offer another tactical context.

In the wide configuration, offensive teams took advantage of the higher field width to perform more crosses from exterior channels of the field and wide subspaces of the opponent. Probably due to the higher frequency of crosses, more headers were found in this configuration. In this line, Frencken et al. [33] suggested that the availability of more lateral space at wider pitches offers players the opportunity to move into these regions, increasing teams’ lateral displacement. In this manner, making the field wider seems to increase the importance of crossing and heading to create GSO.

Our study agrees with previous studies [19,26] in suggesting that altering the length and width of the pitch influences players’ tendencies to explore along the goal-to-goal and lateral-to-lateral axes. This modulation of the pitch configuration adds variability to the SSG and promotes the players’ tactical exploration and movement variability, which promotes the emergence of different tactical solutions to create GSO.

### Limitations and Practical Applications

This study has several limitations. Firstly, the fact of only using observational methodology may not capture the entire complexity of soccer actions and interactions, as previous studies based on ecological models have claimed [46,47,48]. Secondly, this study did not measure collective and positional variables (team width and length, centroid distance, etc.), physical or physiological variables (heart rate, distance covered, accelerations, decelerations, etc.), or the accumulated training load, as other similar studies did [19,26,33,45,49]. Finally, this study only focused on offensive dimensions, while the possible effects of different pitch configurations on the defensive playing tactics were not analyzed.

Nevertheless, this study has important practical applications. Our findings suggest that soccer coaches should consider the manipulation of the pitch configuration to expose players to different spatial constraints and make them explore multiple solutions to create GSO.

## 5. Conclusions

In conclusion, the long and wide configurations produced more counterattacks, higher offensive penetration, and more GSO than the standard format. Regarding the creation of GSO, the wide configuration produced more assists in the form of crosses than the long and standard formats, more utilization of wide subspaces to assist the final player, greater number of headers as the final action, and less assists in the form of passes in behind the defense than the standard configuration.

Thus, the modulation of the spatial constraints by changing the pitch configuration during small sided soccer games produces different tactical demands, which requires players to adapt to the spatial context and implement different tactical solutions to create GSO.

## Figures and Tables

**Table 1:** Design of the small sided games.

Task Constraints	Description
Player number (including goalkeepers)	7 vs. 7
Floaters	1 (playing as a central back)
Area per player (m^2^)	132 m^2^
Time (work: passive recovery)	(5:2 min)
Tactical objective	Offensive: To create goal scoring opportunities and goals
	Defensive: To stay compact in a low block defense to protect the goal
Team systems	Offensive: 1.2.3.2
	Defensive: 1.3.3
Feedback	No direct instructions
Rules	Official soccer rules (including offside). The only exception is that all the restarts are taken as goal kicks (no throw ins, corner kicks, or free kicks)
Pitch configurations	Standard (53 × 38 m)
	Long (63 × 32 m)
	Wide (43 × 47 m)

**Table 5:** Comparison of playing tactics between the three different pitch configurations in the team possessions that led to GS.

Category	Standard	Long	Wide	*p* *	ES ^#^
	Mean ± SD (Median)	Mean ± SD (Median)	Mean ± SD (Median)		
Type of attack					
Counterattack	35.3 ± 35.2 (35.9)	47.8 ± 33.5 (40.0)	25.8 ± 22.1 (33.3)	0.412	0.07
Positional attack	64.7 ± 35.18 (55.5)	52.2 ± 33.5 (60.0)	74.2 ± 22.1(67.2)	0.412	0.07
Pass number	4.5 ± 2.24 (4.9)	4.5 ± 2.7 (3.90)	4.5 ± 1.3 (4.5)	0.667	0.03
Type of start					
Transition play	47.8 ± 41.2 (53.3)	75.4 ± 20.9 (70.8)	55.1 ± 32.1 (60.0)	0.233	0.12
Restart	52.2 ± 41.2 (46.6)	24.6 ± 20.9 (29.1)	44.8 ± 32.1 (40.0)	0.233	0.12
Offensive width					
Maximum width	29.7 ± 37.8 (16.6)	43.7 ± 36.4 (40.0)	48.9 ± 30.73(45.5)	0.636	0.38
Medium width	54.4 ± 41.2 (58.3)	26.1 ± 28.9 (20.00)	40.1 ± 34.5 (40.0)	0.320	0.95
Reduced width	15.8 ± 24.5 (0.0)	30.1 ± 31.8 (22.50)	10.9 ± 14.8 (0.0) ^b^	**0.018**	**0.36**

* Friedman Test. Values in bold indicate significant differences between pitch configurations. ^#^ Effect size calculated using the Kendall’s W (coefficient of concordance; 0.1–0.3 = small effect; 0.3–0.69 = moderate effect; 0.70–1.0 = large effect size ^b^ = significantly different (*p* ˂ 0.05) from the “long” configuration.

**Table 6:** Comparison of the penultimate action between the three different pitch configurations in the team possessions that led to GSO.

Category	Standard	Long	Wide	*p* *	ES ^#^
	Mean ± SD (Median)	Mean ± SD (Median)	Mean ± SD (Median)		
Penultimate action					
Individual play	55.0 ± 43.9 (53.3)	34.4 ± 30.4 (36.6)	37.6 ± 30.2 (33.3)	0.368	0.08
Cross	16.6 ± 28.6 (0)	13.3 ± 20.5 (0)	43.0 ± 25.1 (36.6) ^a,b^	**0.025**	**0.31**
Pass in behind	11.4 ± 18.0 (0)	30.8 ± 31.5 (20.0)	12.8 ± 24.0 (0) ^b^	0.034	0.28
Goal pass	16.9 ± 30.9 (0)	21.4 ± 30.2 (10.0)	6.5 ± 12.1 (0)	0.629	0.39
Assisting player					
Central Back	0 (0)	5.5 ± 16.6 (0.0)	10.6 ± 18.6 (0.0)	0.368	0.14
Full Back	56.7 ± 31.3 (50.0)	37.9 ± 42.4 (20.0)	65.9 ± 33.8 (66.7)	0.432	0.12
Midfielder	24.3 ± 38.2 (0.0)	20.00 ± 33.5 (0.0)	11.4 ± 30.3 (0.0)	0.444	0.12
Forward	19.0 ± 24.4 (0.0)	37.6 ± 43.3 (33.3)	12.1 ± 16.8 (0.0)	0.390	0.13
Assisting Space (width level)					
Central subspaces	56.1 ± 34.0 (50.0)	68.7 ± 20.1 (66.7)	34.7 ± 20.8 (33.3) ^b^	**0.016**	**0.35**
Wide subspaces	43.9 ± 34.0 (50.0)	29.2 ± 22.0 (33.3)	65.3 ± 20.8 (66.7) ^b^	**0.016**	**0.35**
Assisting space (penetration level)					
Subspace behind the defense	19.4 ± 32.4 (0)	27.5 ± 31.7 (25.0)	26.5 ± 24.1 (29.1)	0.704	0.03
Defensive subspace	80.6 ± 32.4 (100)	72.5 ± 31.7 (75.0)	73.5 ± 24.1 (70.8)	0.704	0.03

* Friedman Test. Values in bold indicate significant differences between pitch configurations. ^#^ Effect size calculated using the Kendall’s W (coefficient of concordance; 0.1–0.3 = small effect; 0.3–0.69 = moderate effect; 0.70–1.0 = large effect size. ^a^ = significantly different (*p* ˂ 0.05) from the “standard” configuration. ^b^ = significantly different (*p* ˂ 0.05) from the “long” configuration.

**Table 7:** Comparison of the final action between the three different pitch configurations in the team possessions that led to GSO.

Category	Standard	Long	Wide	*p* *	ES ^#^
	Mean ± SD (Median)	Mean ± SD (Median)	Mean ± SD (Median)		
Final player					
Central Back	5.5 ± 12.9 (0)	7.5 ± 11.7 (0)	2.1 ± 7.2 (0)	0.431	0.07
Full Back	18.3 ± 29.7 (0)	23.0 ± 30.7 (10.0)	7.9 ± 12.9 (0)	0.449	0.07
Midfielder	16.7 ± 38.9 (0)	8.9 ± 16.1 (0)	16.9 ± 22.8 (8.3)	0.446	0.07
Forward	59.4 ± 43.3 (73.3)	60.5 ± 29.8 (60.0)	71.0 ± 25.2 (70.8)	0.452	0.07
Final Space (width level)					
Central subspaces	74.7 ± 32.9 (10.0)	73.6 ± 30.9 (81.6)	74.4 ± 20.2 (75.0)	0.836	0.01
Wide subspaces	25.3 ± 32.9 (90.0)	26.5 ± 30.9 (18.8)	25.5 ± 20.2 (25.0)	0.836	0.01
Final space (penetration level)					
Behind the defense	60.0 ± 37.5 (50.0)	52.16 ± 33.38 (45.0)	53.5 ± 29.1 (50.0)	0.832	0.01
Defensive subspace	40.0 ± 37.6 (50.0)	47.91 ± 33.34 (55.0)	46.5 ± 29.1 (50.0)	0.832	0.01
Last action					
Shot (2 contacts)	76.9 ± 25.1 (83.3)	55.8 ± 38.6 (66.7)	47.2 ± 29.5 (50.0)	0.232	0.12
Shot (1contact)	18.8 ± 24.4 (0)	42.5 ± 40.1 (33.30)	36.1 ± 34.8 (29.1)	0.423	0.07
Header	4.2 ± 14.4 (0)	1.7 ± 5.8 (0)	16.6 ± 18.4 (12.5) ^b^	**0.022**	**0.32**

* Friedman Test. Values in bold indicate significant differences between pitch configurations. ^#^ Effect size calculated using the Kendall’s W (coefficient of concordance; 0.1–0.3 = small effect; 0.3–0.69 = moderate effect; 0.70–1.0 = large effect size. ^b^ = significantly different (*p* ˂ 0.05) from the “long” configuration.

## Data Availability

The data that support the findings of this study are available from the corresponding author, upon reasonable request.
